# Post-surgical Gangrene with Pseudomonas luteola Resulting in Limb Amputation: A Case Review

**DOI:** 10.7759/cureus.3441

**Published:** 2018-10-11

**Authors:** Wallisa Roberts, Carmen Roessler, Petra J Francis, Dolland Noel, Marios Loukas

**Affiliations:** 1 General Surgery, Grenada General Hospital, St. George, GRD; 2 Anatomy, St. George's University, St. George, GRD; 3 Surgery, Grenada General Hospital, St. George, GRD; 4 Internal Medicine, Grenada General Hospital, St. Georges, GRD; 5 Anatomy, St. George's University, St. George's, GRD

**Keywords:** pseudomonas, amputation, gangrene, luteola

## Abstract

Pseudomonas luteola is a rare infective agent with a variable resistance-sensitivity panel. Clinical suspicion and appropriate empiric treatment is necessary for resolution of such infections. We report a case of post-surgical gangrene as a result of Pseudomonas luteola culminating in limb amputation.

## Introduction

*Pseudomonas luteola* (*P. luteola*) is an aerobic, motile Gram-negative rod appearing as light to deep shades of yellow colonies on MacConkey or blood agar. However, it differs from most of the other members of the *Pseudomonas* group, as it is oxidase negative [1]. *P. luteola* has clinical significance as it has been implicated in a variety of life-threatening infections such as: endocarditis, peritonitis, meningitis, septicemia and brain abscesses [2-4]. Moreover, it has also been diagnosed as the causal factor in less common infections such as: endophthalmitis, mediastinal botryomycosis and osteomyelitis [5-7].

*P. luteola* was initially designated to the CDC biogroup Ve-1 group due to its multi-trichous polar flagella [8]. The bacteria underwent several name and genus changes: *Chromobacterium typhiflavum, Chryseomonas polytrichia, *and* Chryseomonas luteola*, until 1997, when Anzai et al. performed a 16S rRNA sequence analysis which revealed the synonymy of the *Chryseomonas, Flavimonas *and* Pseudomonas* groups [9]. We report a case of *P. luteola* isolation from a traumatic wound that later resulted in lower limb amputation.

## Case presentation

**Figure 1:**
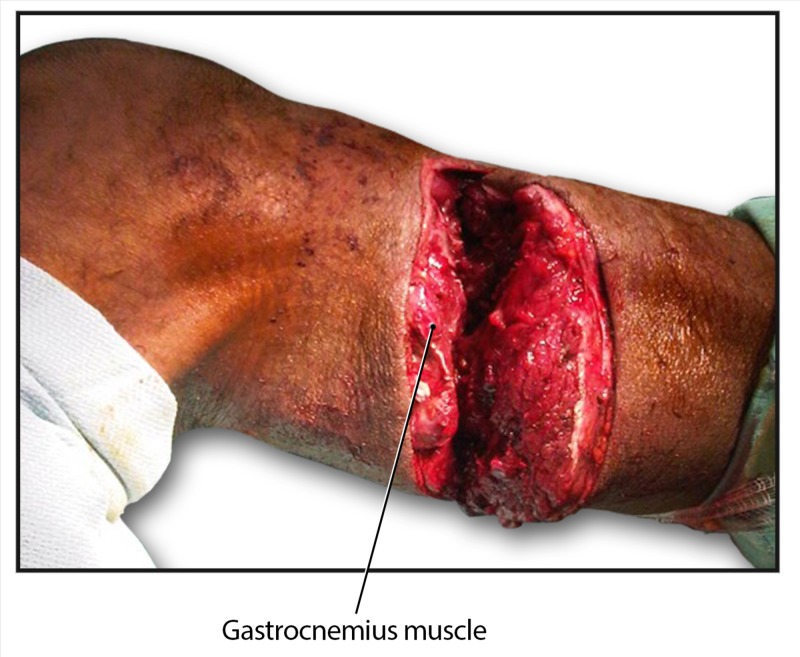
Partial amputation of the patient's right leg secondary to a traumatic injury.

A 43-year-old man was brought to the emergency room with deep machete lacerations to the right forearm, a superficial laceration to the left forearm, and a deep laceration and partial amputation of the right lower leg (Figure 1).

**Figure 2:**
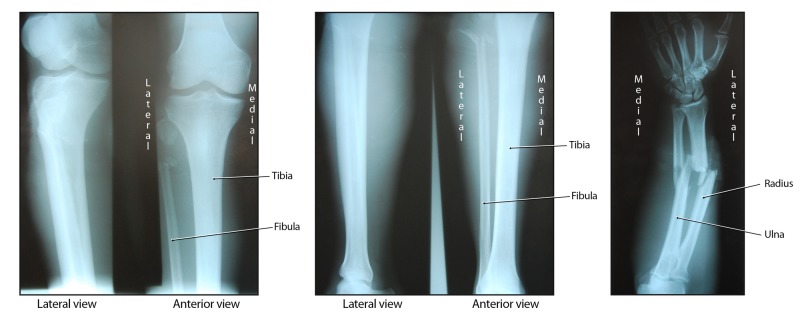
Radiographic images of the patient's polytraumatic presentation.

Radiographs showed complete comminuted and displaced fractures of the right midshaft of the radius and ulna, as well as a comminuted fracture of the left proximal fibula (Figures 2).

A chest radiograph was unremarkable. His past medical history included asthma and a hospital admission for an abscess of the right thumb in 2009.

Immediate care included antibiotic coverage with cloxacillin 500 mg IV every six hours and gentamicin 80 mg IV every eight hours. Intraoperative reconstruction and external fixation of the forearm was performed, as well as reconstruction of the fibula with common peroneal nerve and gastrocnemius muscle repair. Postoperatively, cloxacillin and gentamicin were continued for a total of seven and five days, respectively.

Five days after admission, the patient developed signs of infection in the right lower limb and debridement was performed twice in the operating theatre. Ceftazidime 1 g IV every eight hours and crystapen benzylpenicillin two megaunits IV every six hours were added to the antibiotic regime. Wound swabs were obtained, and Gram stains showed numerous Gram-negative bacilli and some Gram-positive cocci, as well as yeast cells. However, wound cultures grew only *P. aeruginosa* and *P. luteola*. The *P. aeruginosa* was sensitive to amikacin, ceftazidime, ciprofloxacin, gentamicin and tobramycin, but was resistant to carbenicillin. *P. luteola* displayed sensitivity to amikacin, ceftazidime, ciprofloxacin, gentamicin and tobramycin with resistance to ampicillin, augmentin, bactrim and imipenem, and intermediate sensitivity to cefotaxime and ceftriaxone.

**Figure 3:**
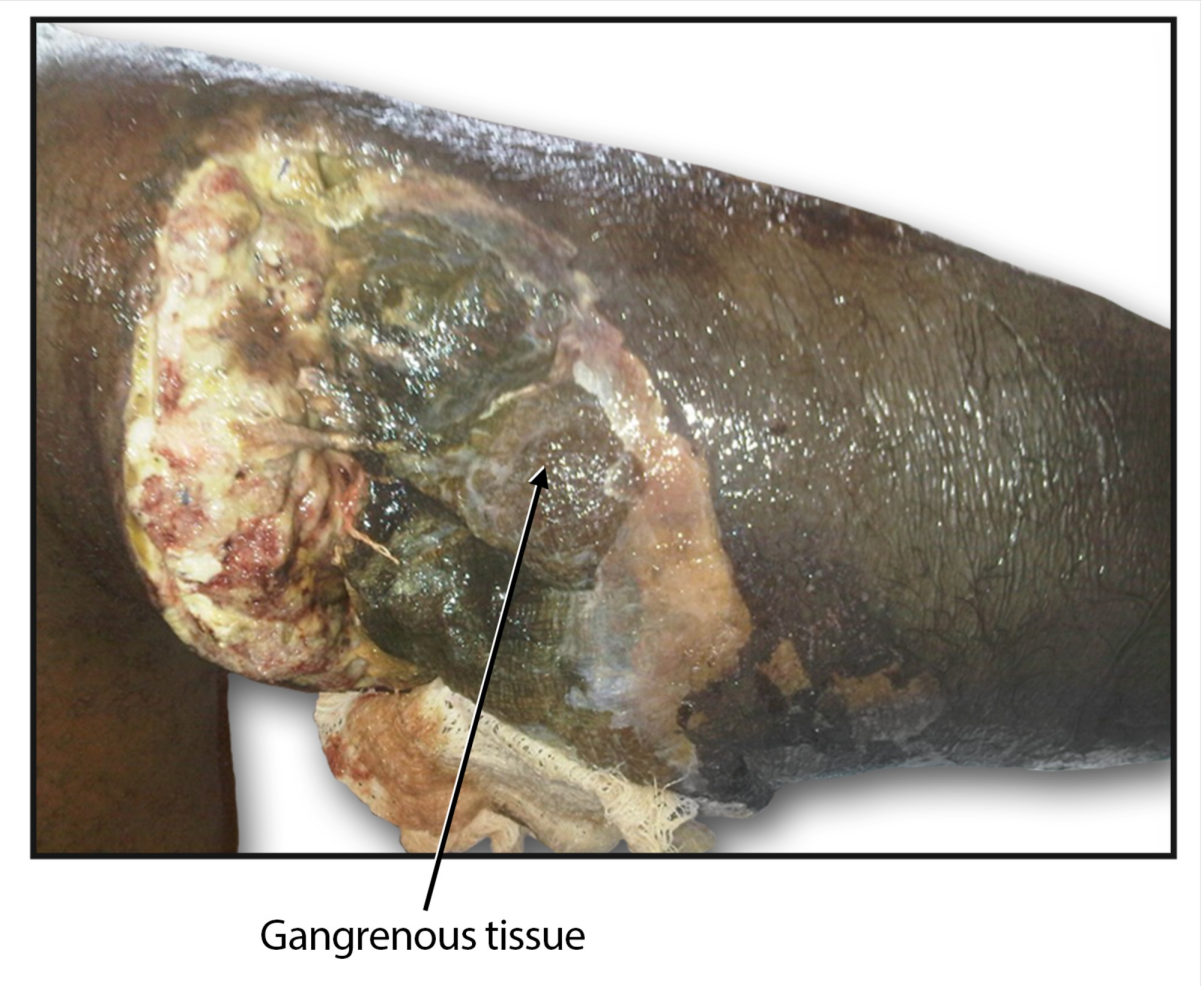
Postoperative complication of gangrene of the right leg.

On hospital day seven, the patient spiked a temperature of 38.1°C and the lower limb showed signs of gangrene (Figure 3); the foot was cold and swollen with loss of sensation, peripheral pulses were absent, and palpable crepitations were present. A diagnosis of gas gangrene was made.

The patient and his family were informed of the available treatment options and together with the medical team, a decision was made to perform an above-knee amputation of the right leg. Post amputation, the patient’s health improved, and the rest of his hospital stay was uneventful. Three months after the trauma incident, the right stump was clean and healing well. The fractures of the right forearm were also healing, and good callus formation was seen.

## Discussion

In the Caribbean and particularly in Grenada, diabetes is usually the leading cause of lower limb amputations [10]. Although *Staphylococcus aureus* is the organism most commonly associated with such infections leading to lower extremity amputations, the *Pseudomonas* genus is also known to cause gangrene resulting in amputation [11]. *P. luteola* has been isolated from both ulcers and osteomyelitis cases, but as far as we are aware, no such cases have resulted in amputation of a limb [6,7,12]. To the best of our knowledge, this is the first reported case of *P. luteola* infection in Grenada, and quite possibly the first case of *P. luteola* to be isolated from a wound that later resulted in amputation.

*P. luteola* is an infrequent human pathogen. The natural habitat of the pathogen has not been determined, but it belongs to a group of bacteria that are usually found in water, soil, and other moist environments [13]. In most clinical cases to date, it has been associated with foreign bodies and indwelling catheters often mandating removal [1], corticosteroid use, and other immuno-compromised states [1, 7,14,15]. In both of the previous reports of *P. luteola*-induced osteomyelitis, patients were healthy with no known underlying medical conditions, and with no reasons to be chronically immuno-compromised. In this case, the patient was a healthy individual, who had not used any steroids, and no long-standing extraneous matter was found to be present in the leg.

As found in our circumstance, in vitro studies have typically demonstrated the susceptibility of *P. luteola* to aminoglycosides and fluoroquinolones such as ciprofloxacin. However, variable resistance has been noted against many β-lactam-containing antibiotics such as penicillins and cephalosporins [12]. This prompted the discovery of blaLUT-1 and other new β-lactamase gene variants by Doublet et al. [13]. It is important to acknowledge that due to its variable resistance pattern, this bacterium should be considered as a possible culprit when infections are un-resolving – particularly in immuno-compromised patients and those with indwelling foreign bodies [1,13]. As was seen in this instance,* P. luteola* can be involved in infections that lead to severe morbidity, and may even result in mortality. In their retrospective study, Bayhan et al. assessed the number of cases of *P. luteola* at a pediatric hospital [16]. They found that the majority of cases of *P. luteola* (85.7%, 6/7 patients) were hospital acquired and that nosocomial infection by the pathogen tended to present as bacteremia. From their sensitivity-resistance panel, they suggested that carbapenems were the most appropriate agent of choice for nosocomial *P. luteola* as all their isolates were susceptible to this class of drug, even though currently no standardized regimen exists. *P. luteola* has a relapsing/chronic course when the right antibiotic treatment is not selected [7]. Therefore, the authors feel a consensus regarding imperative therapy is needed. Thus, from our preliminary review of the sensitivity and resistance reported in the case studies [2,3,7,12,14-19] examined (Table 1), the authors would like to suggest that a suitable initial therapy when this organism is encountered might be a beta-lactam and aminoglycoside, specifically imipenem and amikacin.

**Table 1:** Preliminary overview of the resistance and sensitivity panel of P. luteola based on case reports.

Study	Antibiotic Sensitivity/Resistance							
	Amikacin	Gentamicin	TSX	Meropenem	Tobramycin	Ceftazidime	Ciprofloaxin	Carbenicillin
Bayhan et al. [16]	+	+	+	+				
Connor et al. [2]	+	+	+		+			-
Ghosh [14]			+				+	
Tsakris et al. [12]	+	+	-			+	+	
Jayagopal et al. [7]								
De et al. [17]	+					-	+	
Berger et al. [15]	+	+						
Chihab et al., case one [3]	+	-	-				+	
Chihab et al., case two [3]			-				+	
Yetkin et al. [18]		+	+	+	+	+	+	
Kilic et al. [19]			+	+			+	
Study	Antibiotic Sensitivity/Resistance							
	Cephalothin	Cefoxitin	Cefamandole	Ceftriaxone	Cefazolin	Cefuroxime	Ceftazidime	Piperacillin
Bayhan et al. [16]								
Connor et al. [2]	-	-	-					
Ghosh [14]							+	
Tsakris et al. [12]				-		-		+
Jayagopal et al. [7]								
De et al. [17]				-				
Berger et al. [15]								
Chihab et al., case one [3]				-			-	
Chihab et al., case two [3]				+			+	
Yetkin et al. [18]				+		+		+
Kilic et al. [19]				+	+		+	
Study	Antibiotic Sensitivity/Resistance							
	Piperacillin/tazobactam	Cefepime	Imipenem	Pefloxacin	Tobramycin	Ampicillin	Amoxicillin	Amoxiclav
Bayhan et al. [16]								
Connor et al. [2]								
Ghosh [14]	+							
Tsakris et al. [12]		+	+	+	+	-		-
Jayagopal et al. [7]								
De et al. [17]	-		+			-		-
Berger et al. [15]								
Chihab et al., case one [3]			+				-	
Chihab et al., case two [3]			+				-	
Yetkin et al. [18]						-		
Kilic et al. [19]			+			+		+
Study	Antibiotic Sensitivity/Resistance							
	Cephalothin	Cefoxitin	Norfloaxin	Nalidixic acid	Oxytetracycline	Colistin		
Bayhan et al. [16]						-		
Connor et al. [2]								
Ghosh [14]								
Tsakris et al. [12]	-	-						
Jayagopal et al. [7]					+			
De et al. [17]								
Berger et al. [15]						+		
Chihab et al., case one [3]						+		
Chihab et al., case two [3]						+		
Yetkin et al. [18]								
Kilic et al. [19]						+		
Study	Antibiotic Sensitivity/Resistance							
	Ofloxacin	Cefotaxime	Doxycycline	Netilmicin	Ureidopenicillin	Fluoroquinolone	Tetracycline	Cefoperasone
Bayhan et al. [16]								
Connor et al. [2]								
Ghosh [14]								
Tsakris et al. [12]								
Jayagopal et al. [7]								
De et al. [17]		-						
Berger et al. [15]		+					+	
Chihab et al., case one [3]	+	-	+	+				
Chihab et al., case two [3]	+	+	+					
Yetkin et al. [18]								
Kilic et al. [19]	+	+						
Study	Antibiotic Sensitivity/Resistance							
	Tigecycline	Azlocillin	Kanamycin	Mezlocillin	Streptomycin	Tetracycline	Cephalothin	Chlorampenical
Bayhan et al. [16]								
Connor et al. [2]								
Ghosh [14]								
Tsakris et al. [12]								
Jayagopal et al. [7]								
De et al. [17]								
Berger et al. [15]		+	+	+	+	+		
Chihab et al., case one [3]								
Chihab et al., case two [3]							-	
Yetkin et al. [18]								
Kilic et al. [19]				+				
Study	Antibiotic Sensitivity/Resistance							
	Polymyxin B	Penicillin G	Novobiocin	Vibriostatic compound O/129	Fosfomycin	Moxalactam	Aztreonam	Cefaperazone
Bayhan et al. [16]								
Connor et al. [2]								
Ghosh [14]								
Tsakris et al. [12]								
Jayagopal et al. [7]								
De et al. [17]								
Berger et al. [15]								
Chihab et al., case one [3]								
Chihab et al., case two [3]								
Yetkin et al. [18]							+	
Kilic et al. [19]								
Study	Antibiotic Sensitivity/Resistance							
	Cefoperazone-sulbactam	Cephalexin	Cephadroxil					
Bayhan et al. [16]								
Connor et al. [2]								
Ghosh [14]								
Tsakris et al. [12]								
Jayagopal et al. [7]								
De et al. [17]								
Berger et al. [15]								
Chihab et al., case one [3]								
Chihab et al., case two [3]	+							
Yetkin et al. [18]								
Kilic et al. [19]								
Legend								
+	Sensitive							
-	Resistant							
	Unavailable							

This drug combination was also chosen due to the fact that *P. luteola* tends to present with septicemia [5]. Furthermore, Imipenem was chosen as an appropriate empiric drug, despite our isolate resistance to it, as generally from the preliminary panel, *P. luteola* tends to be sensitive to Imipenem. Tamma et al., in their study, showed that for severely ill patients secondary to gram-negative infections, combination therapy resulted in better survivability than monotherapy in certain subgroups [20].

## Conclusions

In summary, due to its serious clinical implications, as well as the fact that it has been identified as a nosocomial infection, it is imperative that microbiologists and physicians alike are aware of *P. luteola*, particularly due to its multidrug resistance variability, and the chronic/relapsing course that is seen when the organism is not treated with the correct antibiotics. We therefore suggest a beta-lactam/aminoglycoside combination of amikacin/imipenem. We recognize that further research is needed to elucidate a definitive imperative treatment.
